# Time-of-day-dependent expression of purinergic receptors in mouse suprachiasmatic nucleus

**DOI:** 10.1007/s00441-017-2634-8

**Published:** 2017-05-26

**Authors:** Julian Lommen, Anna Stahr, Marc Ingenwerth, Amira A. H. Ali, Charlotte von Gall

**Affiliations:** 1 Institute of Anatomy II, Medical Faculty, Heinrich-Heine-University, Moorenstrasse 5, 40225 Düsseldorf, Germany; 2 Institute of Pathology, University of Duisburg-Essen, University Hospital Essen, Hufelandstrasse 55, 45147 Essen, Germany

**Keywords:** Purinergic signalling, P2X, P2Y, Circadian rhythm, Suprachiasmatic nucleus

## Abstract

Purinergic P2X and P2Y receptors are involved in mediating intercellular signalling via purines such as adenosine triphosphate (ATP). P2X and P2Y receptors have been implicated in numerous body functions including learning, memory and sleep. All of these body functions show time-of–day-dependent variations controlled by the master circadian oscillator located in the suprachiasmatic nucleus (SCN). Evidence exists for a role of purinergic signalling in intercellular coupling within SCN. However, few studies have been performed on the expression of purinergic receptors in SCN. Therefore, we analyse the expression of seven P2X (P2X1–7) and eight P2Y (P2Y1–2, 4, 6, 11–14) receptors in mouse SCN and address their time-of-day-dependent variation by using immunohistochemistry and real-time polymerase chain reaction. At the early light phase, P2X and P2Y receptors show a low to moderate, homogenously distributed immunoreaction throughout SCN. P2Y13 reveals strong immunoreaction in fibres within the core region of SCN. From the fifteen analysed P2 receptors, seven exhibit a time-of-day-dependent variation in SCN. P2X1 immunoreaction is very low in the early light phase with a minor increase at the end of the dark phase. P2X4 immunoreaction strongly increases during the dark phase in soma cells in the core region and in a dense network of fibres in the shell region of SCN. P2X3 immunoreaction is moderately elevated during the dark phase. Conversely, immunoreaction for P2Y2, P2Y12 and P2Y14 moderately increases at the early light phase and P2Y6 immunoreaction displays a moderate increase at the mid-light phase. Thus, this study demonstrates a time-of-day-dependent variation of P2 receptors in mouse SCN.

**Electronic supplementary material:**

The online version of this article (doi:10.1007/s00441-017-2634-8) contains supplementary material, which is available to authorized users.

## Introduction

Purinergic (P) receptors play a role in cell-cell communication mediated by adenosine (P1 receptors) or ATP (P2 receptors; Burnstock and Kennedy 1985). P2 receptors represent ligand-gated ionotropic (P2X) or G-protein-coupled metabotropic (P2Y) receptors, which are further divided into subclasses (P2X1–7 and P2Y1–2, P2Y4, P2Y6 and P2Y11–14) (Abbracchio and Burnstock 1994; Burnstock 2007). P2X receptors are activated by ATP, leading to the opening of K^+^, Na^+^ and Ca^2+^ permeable channels and subsequently to increased intracellular Ca^2+^. P2Y receptors are activated by ATP/ADP, UTP/UDP and UDP-glucose/galactose. The activation of P2Y1, 2, 4, 6 and 11 results in the release of Ca^2+^ from the endoplasmic reticulum, whereas the activation of P2Y12–14 decreases the intracellular Ca^2+^ concentration. This modulation of intracellular Ca^2+^ levels affects neuronal plasticity (Oliveira et al. 2016). ATP and its metabolites are co-transmitters in the brain and P2 receptors are abundantly present in the brain on both neurons and glial cells (Burnstock 2013). P2X receptors mediate fast synaptic transmission and P2Y receptors are involved in slow synaptic transmission (Illes and Ribeiro 2004a, b; Norenberg and Illes 2000; Robertson et al. 2001). Purinergic signalling has been implicated in brain development (Oliveira et al. 2016) and functions including learning and memory, locomotor and feeding behaviour and sleep (Burnstock 2013). Importantly, all of these body functions show time-of–day-dependent variations controlled by the circadian system. The central pacemaker of the mammalian circadian rhythm is located in the suprachiasmatic nucleus (SCN) of the anterior hypothalamus (Moore and Eichler 1972). The SCN controls rhythms in physiology and behaviour by providing rhythmic output projections to other hypothalamic regions. Although circadian rhythms persist in the absence of rhythmic cues, they can be entrained to the environmental time by light received through the eyes. At the cellular level, each SCN neuron represents a circadian oscillator with a molecular clockwork composed of transcriptional/translational feedback loops of clock genes (Reppert and Weaver 2002) controlling rhythms in the expression of output genes such as vasopressin (Jin et al. 1999). However, at the tissue level, a coherent circadian oscillation of the SCN requires the coupling of its constituent cellular oscillators (Welsh et al. 2010). Moreover, the SCN is composed of two subdivisions, namely the ventrolateral part (core region), which receives direct input from retinal ganglion cells via the retinohypothalamic tract and the dorsomedial part (shell region), which sends projections to other brain regions (Moore and Silver 1998; Moore et al. 1995, 2002). The core region co-localizes with gamma-amino butyyric acid (GABA) (Moore and Speh 1993) and vasoactive intestinal peptide (VIP) (Moore et al. 2002), which is necessary for coupling between the two subdivisions (Albus et al. 2005). ATP is a signalling molecule that plays a significant role in intercellular communication between astrocytes and neurons and among astrocytes (Halassa and Haydon 2010; Haydon 2001). In the rat SCN, extracellular ATP concentrations show a time-of-day-dependent rhythm that is negatively correlated with electrical and metabolic activity (Womac et al. 2009; Yamazaki et al. 1994). Moreover, cultured astrocytes show persistent circadian rhythms in extracellular ATP accumulation (Womac et al. 2009) dependent on clock genes and inositol triphosphate (IP3) signalling (Marpegan et al. 2011). These data suggest a potential role of rhythmic extracellular ATP levels in intercellular communication within the SCN. In the brain, purinergic receptors are located on astrocytes and neurons (Kanjhan et al. 1999; Vulchanova et al. 1996). In the hypothalamus, purinergic receptors are expressed in the paraventricular nucleus (PVN) and the supraoptic nucleus (SON) (Guo et al. 2009; Kanjhan et al. 1999; Seidel et al. 2006; Vulchanova et al. 1996; Xiang et al. 1998; Yao et al. 2003; Zemkova et al. 2008) and appear to be involved in modulating homeostasis such as body temperature (Gourine et al. 2002) or the release of hormones from magnocellular neurons (Kapoor and Sladek 2000). The rat SCN expresses various *P2X* and *P2Y* transcripts (Bhattacharya et al. 2013; Collo et al. 1996) and proteins (Bhattacharya et al. 2013; Xiang et al. 2006). P2X2 modulates GABAergic inhibitory synaptic transmission in the SCN, whereas P2X7 and P2Y receptors contribute to the ATP-stimulated increase of intracellular calcium levels in SCN cells (Bhattacharya et al. 2013). These findings suggest a role of purinergic receptors in intercellular coupling within the SCN. However, only a few detailed studies have been carried out on the expression of purinergic receptors in the SCN. Therefore, we analyse the expression of P2X and P2Y receptors in the mouse SCN. To address the question of a time-of-day-dependent variation, the purinergic receptors are analysed at various time points during the 24 h cycle.

## Materials and methods

### Experimental animals

All animal procedures were approved by the North Rhine-Westphalia State Agency for Nature, Environment and Consumer Protection, Germany (case number: 84–02.04.2013.A358) and conform to international guidelines for the care and use of animals.

Male C57Bl/6 mice (12–15 weeks old) were used. Mice were housed in standard cages with free access to food and water. Animals were kept under a light-dark cycle of 12 h light and 12 h darkness (light on 6.00 am = zeitgeber time 00 [ZT00]; light off 6.00 pm = ZT12). To analyse time-of-day-dependent variations, six mice per time point were killed every 4 h at ZT02, ZT06, ZT10, ZT14, ZT18 and ZT22, either by decapitation for RNA isolation (*n* = 3) or by transcardial perfusion for immunohistochemistry (*n* = 3).

### Immunohistochemistry

Mice were deeply anaesthetized by using ketamine:xylazine (100 mg:10 mg/kg body weight) and transcardially perfused with 0.9 % NaCl followed by 4 % paraformaldehyde. Brains were isolated and post-fixed in 4 % paraformaldehyde for 24 h and cryoprotected in 20 % sucrose for another 24 h. Brains were sectioned coronally into 30 μm thick slices by using a cryomicrotome (Reichert-Jung). Slices were permeabilized with phosphate-buffered saline (PBS) containing 0.2 % Triton-X100 and incubated with 0.24 % H_2_O_2_ for 30 min at room temperature. After being washed with PBS/0.2 % Triton-X100, slices were preincubated for 1 h with normal rabbit or goat serum. The specific primary antibodies against the various purinergic receptors (see Table 1) were incubated with the slices overnight at 4 °C. The sections were then washed and incubated with the corresponding biotinylated anti-rabbit or anti-goat secondary antibodies for 2 h at room temperature. Slices were further incubated with Vectastain Elite ABC Kit (Vector Laboratories) for 1 h followed by incubation in 3.3`-diaminobenzidine (Sigma-Aldrich) for 10 min. Slices were rinsed with PBS/0.2 % Triton-X 100, mounted on slides, air-dried and coverslipped with Entellan (Merck Millipore).

**Table 1:** Antibodies used

Antibody	Company and product number	Dilution
Rabbit anti-P2X1 (H-100)	Santa Cruz, sc-25,692	1:75
Rabbit anti-P2X2	GeneTex, GTX10266	1:500
Rabbit anti-P2X3 (H-60)	Santa Cruz, sc-25,694	1:50
Goat anti-P2X4 (N-15)	Santa Cruz, sc-15,187	1:50
Goat anti-P2X5 (N-16)	Santa Cruz, sc-15,191	1:25
Rabbit anti-P2X6	LSBio, LS-C94426	1:100
Goat anti-P2X7 (L-20)	Santa Cruz, sc-15,200	1:50
Rabbit anti-P2Y1	Santa Cruz, sc-20,123	1:50
Rabbit anti-P2Y2	NovusBio, NB110–39032	1:100
Rabbit anti-P2Y4	GeneTex, GTX87199	1:750
Rabbit anti-P2Y6	Alomone labs, APR-011	1:100
Rabbit anti-P2Y11	GeneTex, GTX108241	1:300
Rabbit anti-P2Y12 (P-14)	Santa Cruz, sc-27,152	1:50
Rabbit anti-P2Y13	LSBio, LS-C145104	1:250
Rabbit anti-P2Y14	LSBio, LS-C120603	1:250
Goat anti-rabbit	Vector Laboratories, BA-1000	1:500
Rabbit anti-goat	Vector Laboratories, BA-5000	1:500

### Image acquisition and analysis

Photomicrographs were captured under bright-field illumination by using a Keyence BZ9000 microscope (Keyence, Japan). The microscope settings, especially light intensity and exposure time, were kept constant during all image acquisitions. An observer blind to the time point analysed the density of purinergic receptor immunoreaction (Ir) in the SCN by using open source image analysis software (NIH Image J, https://imagej.nih.gov/ij/). The optical density of purinergic receptor Ir in the SCN region was measured above the threshold in the cell-free neuropil. Data are expressed as the percentage of area of the entire SCN covered by Ir of the respective purinergic receptor (means ± SEM).

### Analysis of mRNA expression levels

Mice were killed by isoflurane. An SCN slice between bregma from −0.08 to −1.5 mm was prepared by using an ice-cold stainless-steel adult mouse brain matrix for coronal sections (Zivic Instruments). From this slice, the SCN was bilaterally dissected by using a 1.0-mm inner-diameter stainless-steel punch needle. The dissected SCN tissue was immediately frozen and stored at −80 °C. Total RNA was isolated by using the RNeasy Lipid Tissue Mini Kit (Qiagen) according to the manufacturer’s protocol. cDNA was prepared by using the QuantiTect Reverse Transcription Kit (Qiagen). For cDNA synthesis, an RNA concentration of 250 ng per probe was employed. Water (Gibco, ThermoFisher Scientific) was used for cDNA dilution to 1.25 ng/μl. Primers for the specific gene sequences of all purinergic receptors were generated by means of *NCBI Primer-BLAST* software (http://www.ncbi.nlm.nih.gov/tools/primer-blast/). Quantitative real-time polymerase chain reaction (PCR) for all purinergic receptors and housekeeping genes (see Table 2) was performed by using KAPA SYBR FAST qPCR Kit Master Mix ABI Prism (KAPA Biosystems) in an ABI StepOne Plus Real-Time PCR System (Applied Biosystems) with the following PCR program: activation at 95 °C for 5 min followed by 40 cycles of denaturation at 95 °C for 3 s followed by amplification and annealing at 60 °C for 20 s. A standard curve of purified PCR product was used for measuring total transcript number. Transcript numbers of all other purinergic receptors were adjusted to the transcript number of the housekeeping gene Rn18S. Relative mRNA levels were represented per 1000 Rn18S. The quality of the amplification product was validated by melting curves and agarose gel electrophoreses. Expression of the purinergic receptor P2Y11 was not detectable.

**Table 2:** Primer list

Gene	Forward primer	Reverse primer
P2X1	CATGGGGACAGCTCCTTTGT	GAGTGCAGCCACTGTCATCT
P2X2	CCAAGGCACCCCTCAAGTAG	CTCTGCCCCTTCTCCCAAAG
P2X3	TGCTTCAACCAACCCAGTGT	TAAGAGCCCCTCTTCTCCCC
P2X4	CCTGGCTTACGTCATTGGGT	AAGTGTTGGTCACAGCCACA
P2X5	TCTACTGCCCCATCTTCCGA	ATAGTGTGGGTTGCAGTGGG
P2X6	GCTGCACCATGGACCTACTT	GCTTCAGGTGAGCTGTTCCT
P2X7	GCACGAATTATGGCACCGTC	CCCCACCCTCTGTGACATTC
P2Y1	TTATGTCAGCGTGCTGGTGT	ACGTGGTGTCATAGCAGGTG
P2Y2	TCAAACCGGCTTATGGGACC	GGCAGCTGAGGTCAAGTGAT
P2Y4	GCTCTATCTGTTCACGGGGG	AGGGAGGAAGCAGTTGTTCG
P2Y6	GGGTAGTGTGTGGAGTCGTG	AGCGAGTAGACAGGATGGGT
P2Y12	TGCTGTACACCGTCCTGTTC	CGGCTCCCAGTTTAGCATCA
P2Y13	GCATCAGGTGGTCAGTCACA	GTGGGGCAAAGCAGACAAAG
P2Y14	CCACATTGCCAGAATCCCCT	AGCCGAGAGTAGCAGAGTGA
Rn18S	TTCCTTCCGGGCCTTCTCTA	TTGGCAAATGCTTTCGCTC

### Statistical analysis

Statistical analyses between groups were performed by one-way analysis of variance followed by Tukey’s post hoc test or F-test to compare variances and then an unpaired t-test by using Graph Pad Prism software (Graph Pad Software). Data are presented as means ± SEM. Values of *P* < 0.05 were considered statistically significant.

## Results

### Comparative assessment of P2X receptor Ir in medial hypothalamus

**Fig. 1:**
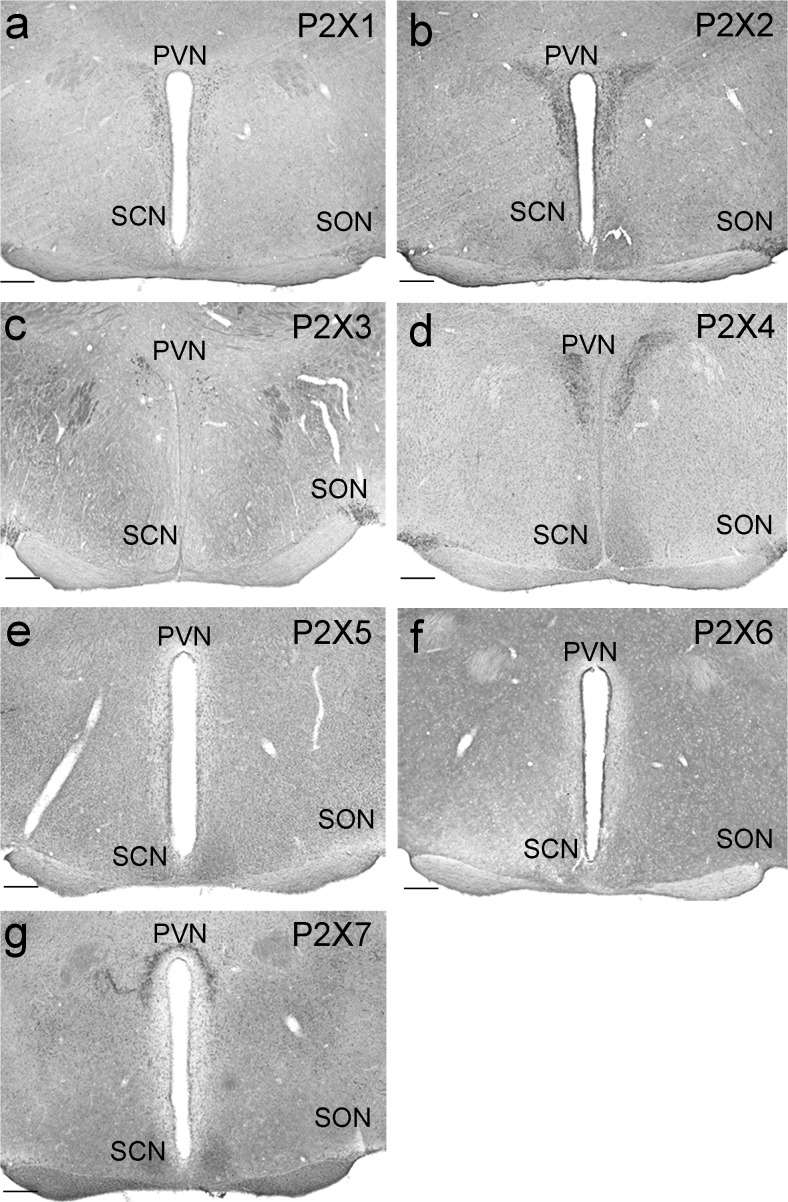
P2X immunoreactivity (Ir) in the medial hypothalamus. Animals were killed 2 h after lights on (zeitgeber time 02 [ZT02]) under a light regime of 12 h light/12 h darkness. Representative photomicrographs (*PVN* paraventricular nucleus of the hypothalamus, *SCN* suprachiasmatic nucleus, *SON* supraoptic nucleus). **a** P2X1 Ir. **b** P2X2 Ir. **c** P2X3 Ir. **d** P2X4 Ir. **e** P2X5 Ir. **f** P2X6 Ir. **g** P2X7 Ir. *Bars* 200 μm

For the comparative assessment of P2X Ir in the medial hypothalamus, the animals were killed during the early light phase (ZT02). P2X1 showed distinct Ir in the PVN and very weak Ir in the SCN (Fig. 1a). P2X2 exhibited strong Ir in both the PVN and the SON and moderate Ir in the SCN (Fig. 1b). P2X3 was strongly stained in cells in the PVN, with strong Ir in the SON and very weak Ir in the SCN (Fig. 1c). P2X4 gave strong Ir in both the PVN and the SON and moderate Ir in the SCN (Fig. 1d). P2X5 showed very weak Ir in both the PVN and the SON and moderate Ir in the SCN (Fig. 1e). Ir for P2X6 was very weak in the PVN, the SON and the SCN (Fig. 1f). P2X7 Ir was distinct in the PVN and moderate in the SCN (Fig. 1g).

### Qualitative assessment of P2X receptor Ir in mouse SCN

**Fig. 2:**
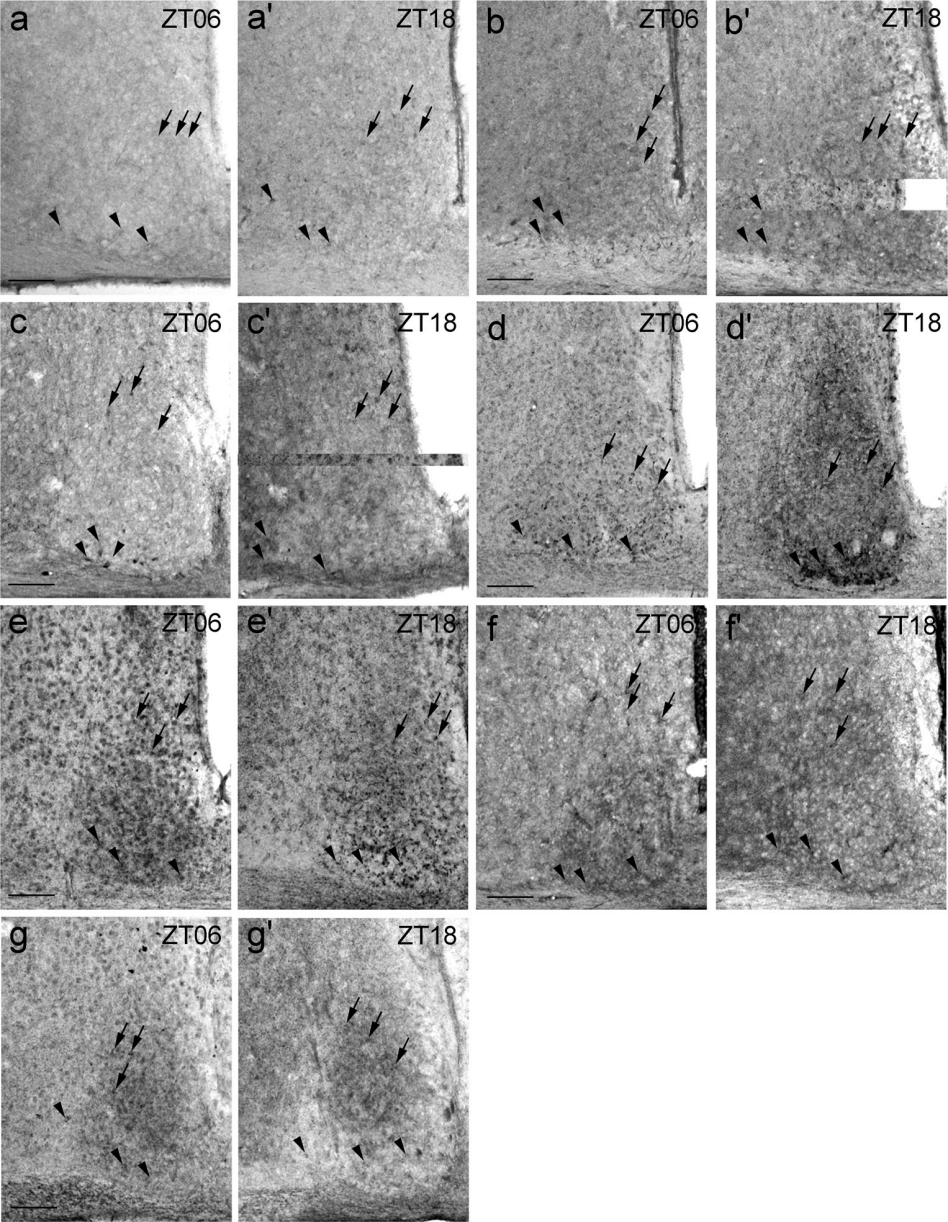
P2X Ir in the SCN. Animals were killed 6 h after lights on (ZT06) and 6 h after lights off (ZT18) under a light regime of 12 h light/12 h darkness. Representative photomicrographs (*black arrows* staining in the shell [dorsomedial] region, *black arrowheads* staining in the core [ventrolateral] region). **a**, **a’** P2X1 Ir. **b**, **b’** P2X2 Ir. **c**, **c’** P2X3 Ir. **d**, **d’** P2X4 Ir. **e**, **e’** P2X5 Ir. **f**, **f’** P2X6 Ir. **g**, **g’** P2X7 Ir. *Bars* 100 μm

For the qualitative assessment of P2X Ir in the core and shell subdivision of the SCN, the animals were killed during the mid-light (ZT06) and mid-dark (ZT18) phase. Generally, P2X Ir was found in both cell bodies and fibres. P2X1 Ir was present in both the core and shell region of the SCN (Fig. 2a, a’). Ir for P2X2 densely labelled neuropil in both the core and shell region of the SCN (Fig. 2b, b’ Supplemental Fig. S1). P2X3 showed Ir in both the core and shell region of the SCN (Fig. 2c, c’). The neuropil within the SCN exhibited stronger P2X3 Ir at ZT18 as compared with ZT06 (Fig. 2c, c’). P2X4 Ir occurred in both the core and shell region of the SCN at ZT06 (Fig. 2d). At ZT18, the Ir for P2X4 was strongly increased, especially in the core region of the SCN and the staining intensity in the neuropil in both SCN subdivisions was strongly increased as compared with that at ZT06 (Fig. 2d, d’). P2X5 Ir was found in both the core and shell region of the SCN and, occasionally, immunoreactive fibres were reminiscent of astrocyte processes (Fig. 2e, e’ Supplemental Fig. S2). P2X6 Ir was revealed in both the core and shell region of the SCN and in a densely labelled neuropil, which was more pronounced in the core region (Fig. 2f, f’). P2X7 Ir in the SCN took the form of a densely labelled neuropil that was more pronounced in the shell region (Fig. 2g, g’).

### Time-of-day-dependent variations in P2X Ir in mouse SCN

**Fig. 3:**
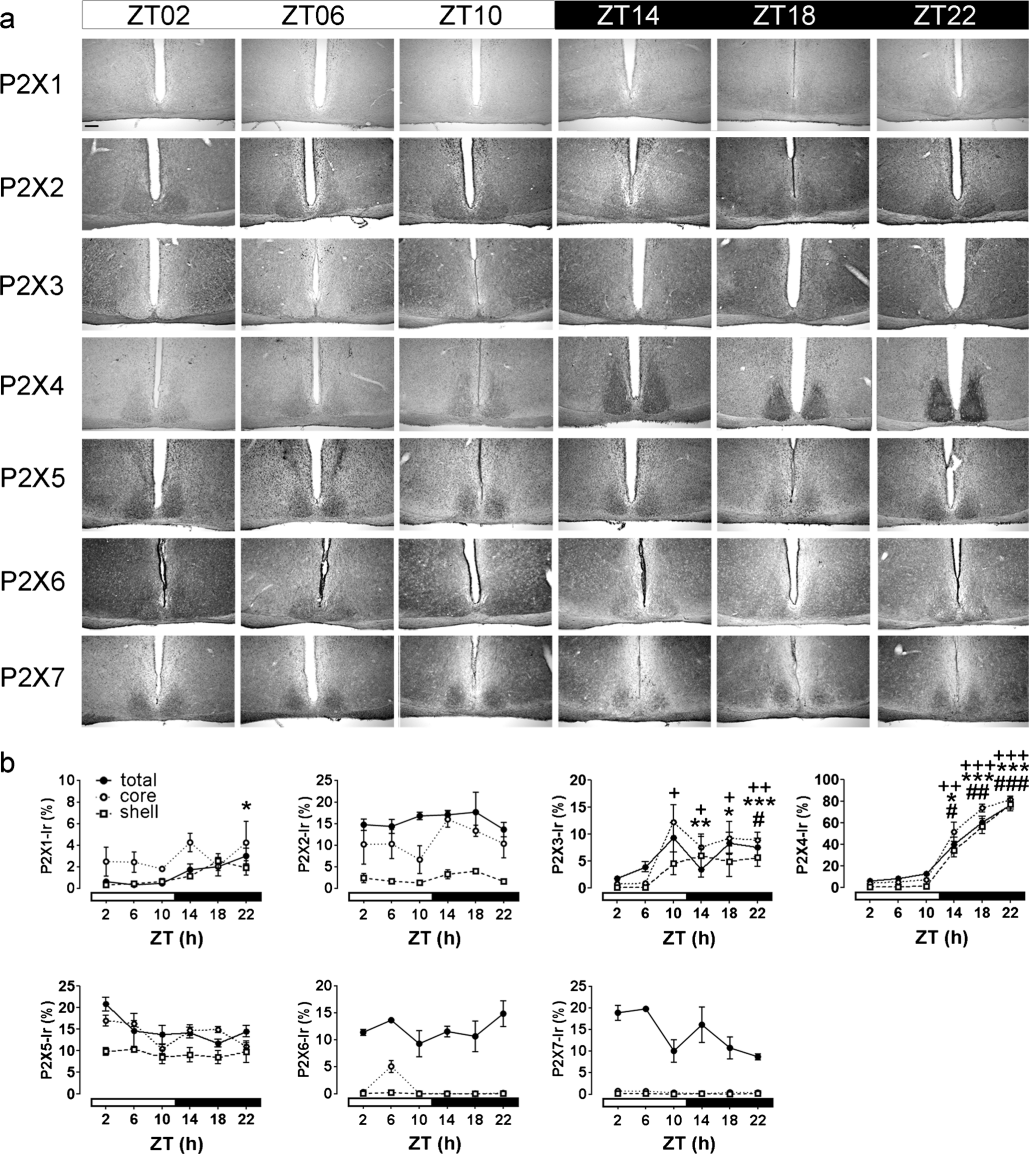
Analysis of time-of-day-dependent variations in P2X Ir in the mouse SCN. Animals were killed at 4-h intervals under a light regime of 12 h light/12 h darkness (ZT00 lights on, ZT12 lights off). **a** Representative photomicrographs of P2X Ir. *Bar* 200 μm. **b** Quantitative analysis of P2X Ir. Data are expressed as means percent of SCN area covered by immunoreaction ± SEM for three animals per time point. The different scales should be noted; *t*-test, *total SCN *P* < 0.05, **total SCN *P* < 0.01, ***total SCN *P* < 0.001; ^+^core region of the SCN *P* < 0.05, ^++^core region of the SCN *P* < 0.01, ^+++^core region of the SCN *P* < 0.001, ^#^shell region of the SCN *P* < 0.05, ^##^shell region of the SCN *P* < 0.01, ^###^shell region of the SCN *P* < 0.001, all compared with ZT02

For the quantitative analyses of P2X Ir in the SCN, the animals were killed at 4-h intervals during the light (ZT02–10) and the dark (ZT14–22) phases. The weak Ir of P2X2, P2X5, P2X6 and P2X7 receptors in the SCN observed at ZT02 did not show significant variations during the 24 h cycle (Fig. 3). In contrast, the very low P2X1 Ir in the SCN at ZT02 slightly but significantly increased during late dark phase (ZT22) in the total SCN (Fig. 3). P2X3 Ir started to increase during the late phase (ZT10) in the core region and during the early night (ZT14) in the shell region of the SCN and stayed elevated in both subdivisions during the dark phase (ZT14–22). P2X4 Ir was low during the light phase and dramatically increased during the dark phase in both SCN subdivisions (Fig. 3).

### Comparative assessment of P2Y receptor Ir in medial hypothalamus

**Fig. 4:**
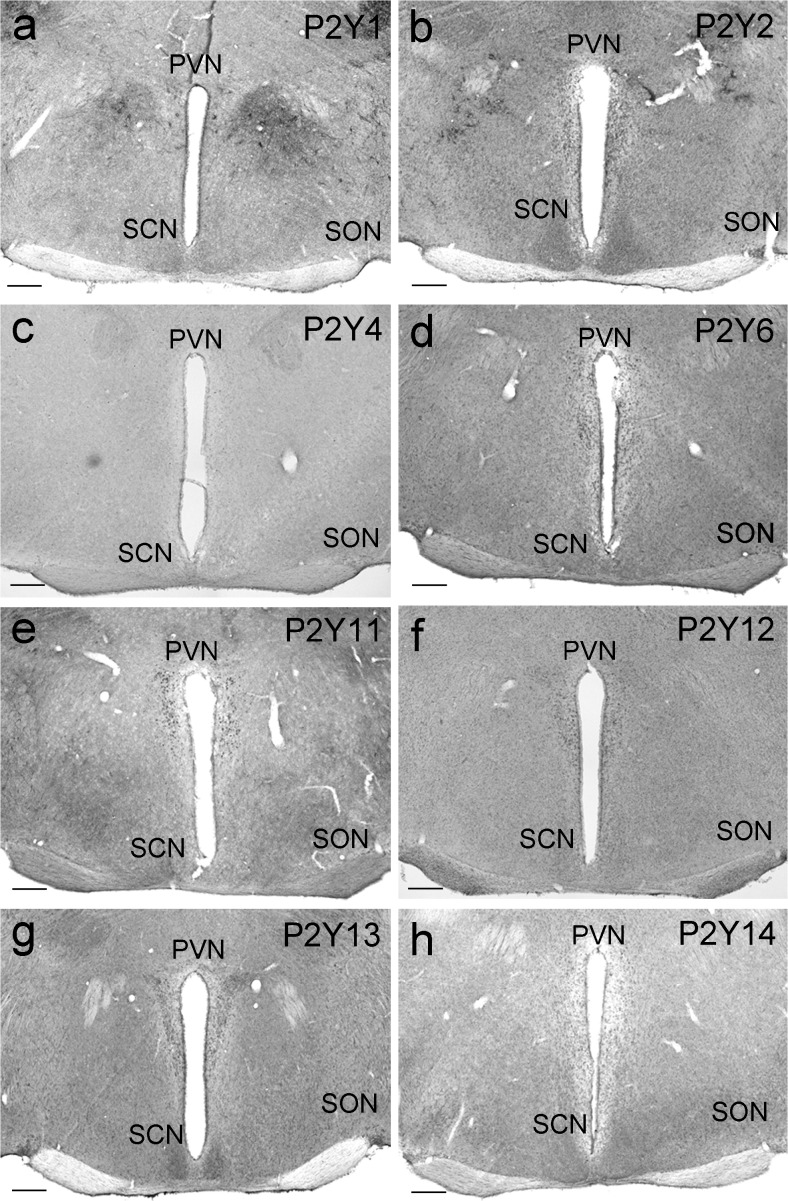
P2Y Ir in the medial hypothalamus. Animals were killed 2 h after lights on (ZT02) under a light regime of 12 h light/12 h darkness. Representative photomicrographs (*PVN* paraventricular nucleus of the hypothalamus, *SCN* suprachiasmatic nucleus, *SON* supraoptic nucleus). **a** P2Y1 Ir. **b** P2Y2 Ir. **c** P2Y4 Ir. **d** P2Y6 Ir. **e** P2Y11 Ir. **f** P2Y12 Ir. **g** P2Y13 Ir. **h** P2Y14 Ir. *Bars* 200 μm

For the comparative assessment of P2Y Ir in the medial hypothalamus, the animals were killed during the early light phase (ZT02). For P2Y1, distinct Ir was found in some cells of the PNV and weak Ir in the SCN (Fig. 4a). P2Y2 showed a moderate Ir in the PVN, the SON and the SCN (Fig. 4b), whereas P2Y4 Ir was very weak in the PVN, the SON and the SCN (Fig. 4c). P2Y6 exhibited moderate Ir in the PVN, the SON and the SCN (Fig. 4d). P2Y11 demonstrated strong Ir in the PVN and very weak Ir in the SCN (Fig. 4e). P2Y12 was present as moderate Ir in the PVN and the SCN (Fig. 4f) and P2Y13 Ir was moderate in the PVN, the SON and the SCN (Fig. 4g). P2Y14 gave weak to moderate Ir in the SCN (Fig. 4h).

### Qualitative assessment of P2Y receptor Ir in mouse SCN

**Fig. 5:**
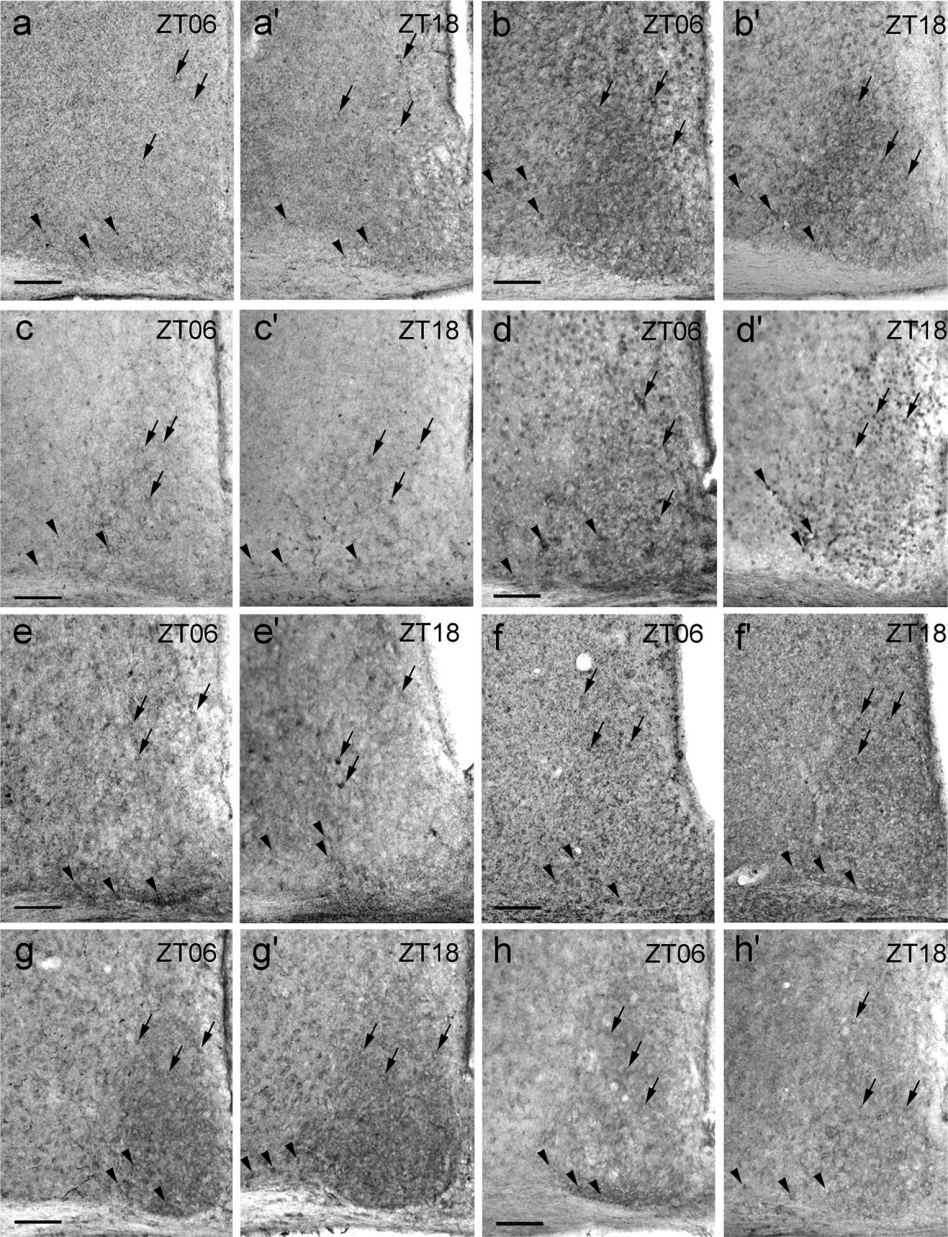
P2Y Ir in the SCN. Animals were killed 6 h after lights on (ZT06) and 6 h after lights off (ZT18) under a light regime of 12 h light/12 h darkness. Representative photomicrographs (*black arrows* staining in the shell [dorsomedial] region, *black arrowheads* staining in the core [ventrolateral] region). **a**, **a’** P2Y1 Ir. **b**, **b’** P2Y2 Ir. **c**, **c’** P2Y4 Ir. **d**, **d’** P2Y6 Ir. **e**, **e’** P2Y11 Ir. **f**, **f’** P2Y12 Ir. **g**, **g’** P2Y13 Ir. **h, h’** P2Y14 Ir. *Bars* 100 μm

For the qualitative assessment of P2Y-Ir in the core and shell subdivision of the SCN, the animals were killed during the mid-light (ZT06) and mid-dark (ZT18) phase. Generally, P2Y Ir was found in both cell bodies and fibres. P2Y1 Ir was present in both the core and shell region of the SCN (Fig. 5a, a’). Some P2Y2 Ir and a densely labelled neuropil were seen in both the core and shell region of the SCN; this was more intense at ZT06 as compared with ZT18 (Fig. 5b, b’). P2Y4 displayed Ir in both the core and shell region of the SCN (Fig. 5c, c’). P2Y6 exhibited cytoplasmic Ir and a densely labelled neuropil in both the core and shell region of the SCN and was more intense at ZT06 than at ZT18 (Fig. 5d, d’). P2Y11 Ir was found in both the core and shell region of the SCN (Fig. 5e, e’). P2Y12 gave Ir in both the core and shell region of the SCN (Fig. 5f, f’). We observed P2Y13 Ir in the SCN, a densely labelled neuropil and occasionally immunoreactive fibres with varicosities reminiscent of nerve fibre terminals in the core region of the SCN (Fig. 5g, g’, Supplemental Fig. S3). P2Y14 showed cytoplasmic Ir in the SCN and a densely labelled neuropil (Fig. 5h, h’).

### Time-of-day-dependent variations in P2Y Ir in mouse SCN

**Fig. 6:**
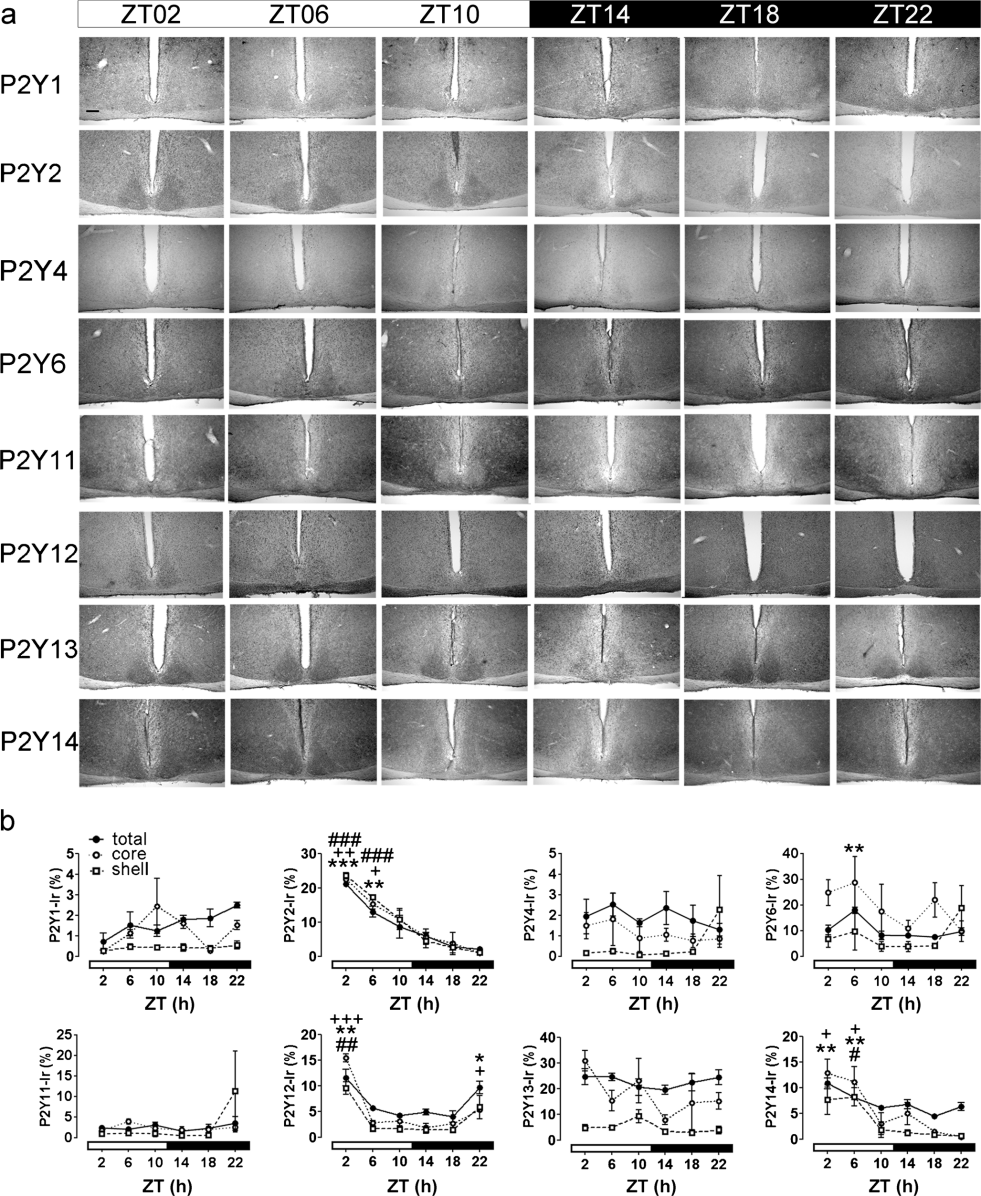
Analysis of time-of-day-dependent variations in P2Y Ir in the mouse SCN. Animals were killed at 4-h intervals under a light regime of 12 h light/12 h darkness (ZT00 = lights on; ZT12 = lights off). **a** Representative photomicrographs of P2Y Ir. *Bar* 200 μm. **b** Quantitative analysis of P2Y Ir. Data are expressed as means percent of SCN area covered by immunoreaction ± SEM for three animals per time point. The different scales should be noted; *t*-test, *total SCN *P* < 0.05, **total SCN *P* < 0.01, ***total SCN *P* < 0.001; ^+^core region of the SCN *P* < 0.05, ^++^core region of the SCN *P* < 0.01, ^+++^core region of the SCN *P* < 0.001, ^#^shell region of the SCN *P* < 0.05, ^##^shell region of the SCN *P* < 0.01, ^###^shell region of the SCN *P* < 0.001, all compared with ZT18

For the quantitative analyses of P2Y Ir in the SCN, the animals were killed in 4-h intervals during the light (ZT02–ZT10) and the dark (ZT14–ZT22) phase. The weak Ir of P2Y1, P2Y4 and P2Y11 receptors in the SCN observed at ZT02 did not show significant variations during the 24 h cycle (Fig. 6). Consistently, the moderate Ir of P2Y13 in the SCN observed at ZT02 did not show significant variations during the 24 h cycle (Fig. 6). P2Y2 Ir was significantly elevated at ZT02 and ZT06 in both the core and the shell region of the SCN (Fig. 6). P2Y6 Ir was significantly elevated at ZT06 in the total SCN (Fig. 6). P2Y12 Ir was significantly elevated at ZT02 in both subdivisions of the SCN and at ZT22 only in the core region of the SCN (Fig. 6). P2Y14 Ir was significantly elevated at ZT02 and ZT06 in the core and at ZT06 in the shell region of the SCN (Fig. 6).

### mRNA expression profile of purinergic receptors in mouse SCN

**Fig. 7:**
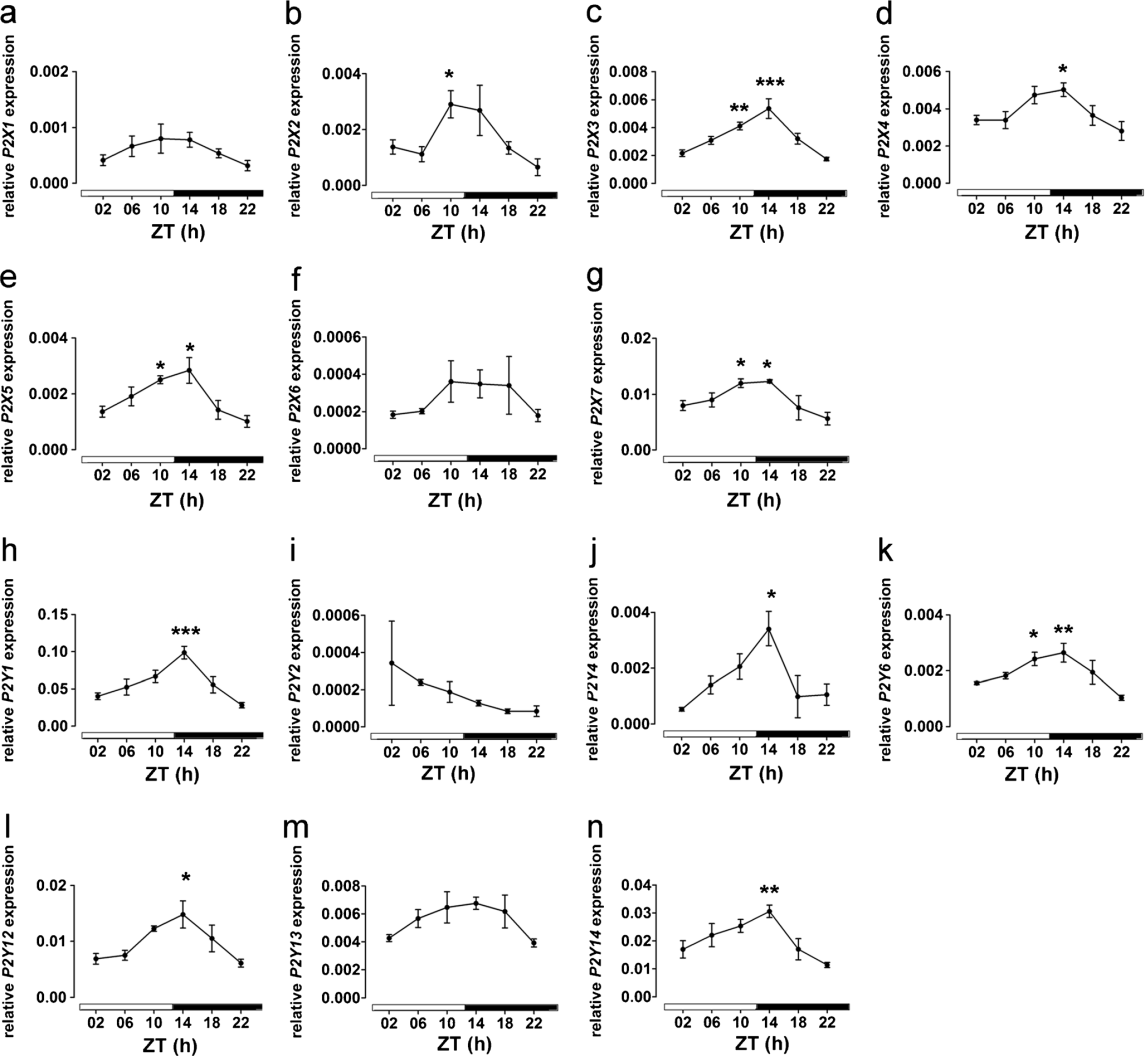
Analysis of time-of-day-dependent variations in *P2X* and *P2Y* gene expression in the mouse SCN. Animals were killed at 4-h intervals under a light regime of 12 h light/12 h darkness (ZT00 = lights on; ZT12 = lights off). **a** Relative *P2X1* mRNA expression profile. **b** Relative *P2X2* mRNA expression profile. **c** Relative *P2X3* mRNA expression profile. **d** Relative *P2X4* mRNA expression profile. **e** Relative *P2X5* mRNA expression profile. **f** Relative *P2X6* mRNA expression profile. **g** Relative *P2X7* mRNA expression profile. **h** Relative *P2Y1* mRNA expression profile. **i** Relative *P2Y2* mRNA expression profile. **j** Relative *P2Y4* mRNA expression profile. **k** Relative *P2Y6* mRNA expression profile. **l** Relative *P2Y12* mRNA expression profile. **m** Relative *P2Y13* mRNA expression profile. **n** Relative *P2Y14* mRNA expression profile. Data are expressed as means ± SEM of three animals per time point. One-way analysis of variance, *P < 0.05, **P < 0.01, ***P < 0.001, all compared with ZT22

In the mouse SCN, the relative mRNA expression levels of purinergic receptors followed the order: P2Y1 > P2Y14 > P2Y12 > P2X7 > P2Y13 > P2X3 > P2X4 > P2X2 = P2Y4 > P2X5 = P2Y6 > P2X1 > P2X6 = P2Y2 (Fig. 7). Among the detectable purinergic receptors, the following 10 showed a time-of-day-dependent variation in relative mRNA expression: P2X2, P2X3, P2X4, P2X5, P2X7, P2Y1, P2Y4, P2Y6, P2Y12 and P2Y14 (Fig. 7). All of these rhythmically expressed purinergic receptors showed the trough expression level at the end of the dark phase (ZT22) and the peak expression level at the early dark phase (ZT14) (Fig. 7).

## Discussion

The major finding of this study is that purinergic receptors and especially P2X4 show a time-of-day-dependent variation in the intensity of Ir in the SCN circadian pacemaker. P2X4 exhibits strong Ir during the dark phase in somata of the core region of the SCN, which receives dense retinal projections and is responsible for light-entrainment and in the neuropil of the shell region of the SCN, which is responsible for autonomous clock function and rhythmic output generation (Moore et al. 2002). This suggests a role for P2X4 in modulating retinal input and communication between the core and the shell region.

In this study, the Ir of seven subtypes of P2X receptor (P2X1–7) and eight subtypes of P2Y receptor (P2Y1–2, P2Y4, P2Y6 and P2Y11–14) and their respective mRNA expression levels were analysed in the mouse SCN at various time points under conditions of 12 h light/12 h dark. During the early light phase, all of these purinergic receptors gave low to moderate Ir. This is consistent with the low Ir of P2X2 observed in the rat SCN (Bhattacharya et al. 2013). We also found that the Ir of most purinergic receptors was homogeneously distributed in the core and the shell region of the SCN. This is consistent with the findings of Bhattacharya et al. (2013) who showed that ATP acts on presynaptic P2X receptors to stimulate GABA release from the core region to the shell region of the SCN. Only P2X7 Ir was more pronounced in the shell region. However, P2X7 and P2Y12 receptors contribute to the ATP-stimulated regulation of intracellular calcium levels but are not involved in modulating ATP-stimulated GABA release in SCN cells (Bhattacharya et al. 2013). Interestingly, P2Y13 Ir was present in nerve-ending-like fibres within the optic chiasm and the core region of the SCN. The projection of the retina to the SCN is important for entrainment of the circadian SCN pacemaker to the environmental light/dark conditions by glutamatergic (Castel et al. 1993) and PACAP-ergic (Hannibal et al. 1997) neurotransmission. Glutamate shifts the phase of the SCN pacemaker during the nighttime (Ding et al. 1994, 1997), whereas this effect mediated by PACAP is restricted to the daytime. Thus, we are tempted to speculate that purinergic receptors are involved in modulating neurotransmission from the retinohypothalamic tract. This hypothesis is consistent with the role of P2 receptors in modulating the release of various neurotransmitters (Bhattacharya et al. 2013; Kapoor and Sladek 2000; Mori et al. 1992).

From the fifteen analysed P2 receptors, seven showed a time-of-day-dependent variation. P2X1 Ir showed a minimal but significant increase at the late dark phase. Ir for P2X3 and P2X4 exhibited a significant increase during the dark phase. During the dark phase, strong P2X4 Ir was present in the somata of cells in the core region of the SCN and in the neuropil of the shell region of the SCN. This suggests a role for P2X4 in the postsynaptic modulation of dark-signal-dependent retinal input and presynaptic modulation of GABA release from the core region to the shell region of the SCN. In contrast, P2Y2, P2Y12 and P2Y14 showed a peak during the early light phase in the core region of the SCN, suggesting that light-dependent retinal input modulates the level of these receptors. The increase of Ir for P2Y12 and P2Y14 in the shell region is delayed by 4 h compared with that of the core region, presumably as a consequence of intra-SCN neuronal coupling.

In contrast to the antiphasic peaks in the Ir rhythms of P2X and P2Y, the gene expression levels of *P2X* and *P2Y* receptors showed coherent peaks at the late light phase/early night phase. Thus, the time-of-day-dependent variations in Ir for P2X and P2Y do not reflect transcriptional regulation. Importantly, in the rat SCN, extracellular ATP shows a time-of-day-dependent rhythm with a peak at the mid-dark phase (Womac et al. 2009). Thus, the increase in P2X3 and P2X4 during the dark phase is coincident with the extracellular ATP accumulation in the SCN and precedes the moderate increase in P2Y2, P2Y12, P2Y14 and P2Y6. Ectonucleotidases, which show a circadian rhythm in various brain regions (Homola et al. 2016), rapidly hydrolyze extracellular ATP to ADP, AMP and adenosine (Yegutkin 2008), a process that terminates P2 activation and prevents receptor desensitization (Junger 2011). The increase in P2Y protein expression during the early to mid-light phase might be a compensatory mechanism in response to the decline in agonist levels.

In conclusion, this study demonstrates a time-of-day-dependent variation of P2 receptors in the mouse SCN and suggests a potential role of P2 signalling, in particular P2X4, in light entrainment and coupling between the SCN subdivisions.

## Electronic supplementary material

Fig. S1 Representative microphotograph of P2X2 immunoreaction in the mouse SCN at high magnification. Mouse was killed at ZT02 (*arrows* P2X2-immunoreactive astrocyte-processes-like fibres). *Bar* 25 μm (GIF 2110 kb).

High Resolution Image (TIFF 1375 kb).

Fig. S2 Representative microphotograph of P2X5 immunoreaction in the mouse SCN at high magnification. Mouse was killed at ZT02 (*arrows* P2X5-immunoreactive astrocyte-processes-like fibres). *Bar* 25 μm (GIF 2246 kb).

High Resolution Image (TIFF 1376 kb)

Fig. S3 Representative microphotograph of P2Y13 immunoreaction in the mouse SCN at high magnification. Mouse was killed at ZT02 (*arrows* P2Y13-immunoreactive nerve-ending-like fibres in the core region of the SCN and the optic chiasm [*oc*]). *Bar* 100 μm (GIF 1658 kb).

High Resolution Image (TIFF 19082 kb).
