# Supplementary figures and images for: Time-of-day-dependent expression of purinergic receptors in mouse suprachiasmatic nucleus
Source: Cell Tissue Res. 2017 May 26;369(3):579–90. doi: 10.1007/s00441-017-2634-8 (PMC5579179; doi:10.1007/s00441-017-2634-8)

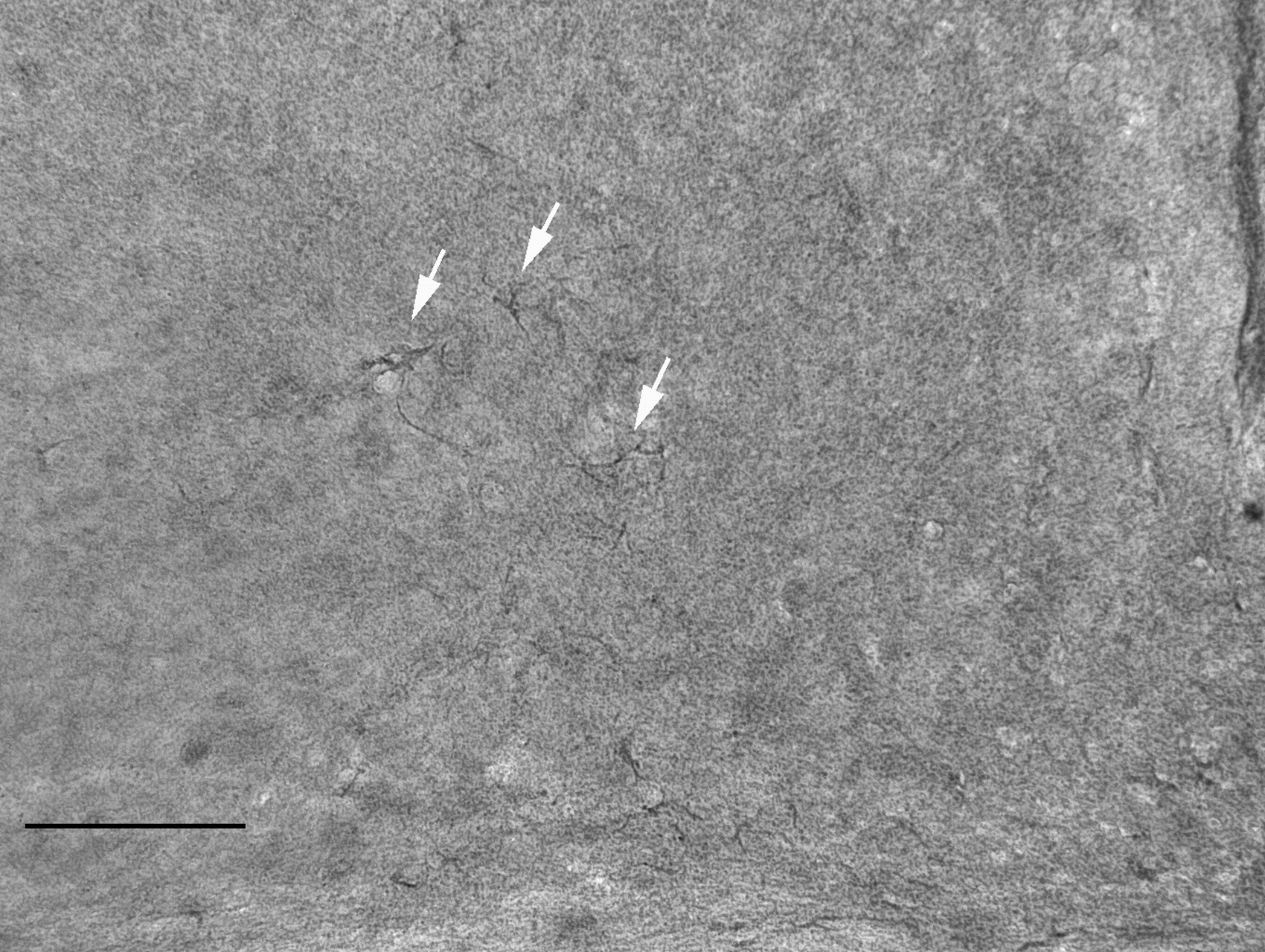

Supplement: Supplementary file 1 — Representative microphotograph of P2X2 immunoreaction in the mouse SCN at high magnification. Mouse was killed at ZT02 (arrows P2X2-immunoreactive astrocyte-processes-like fibres). Bar 25 μm (GIF 2110 kb). [file 441_2017_2634_Fig8_ESM.gif]

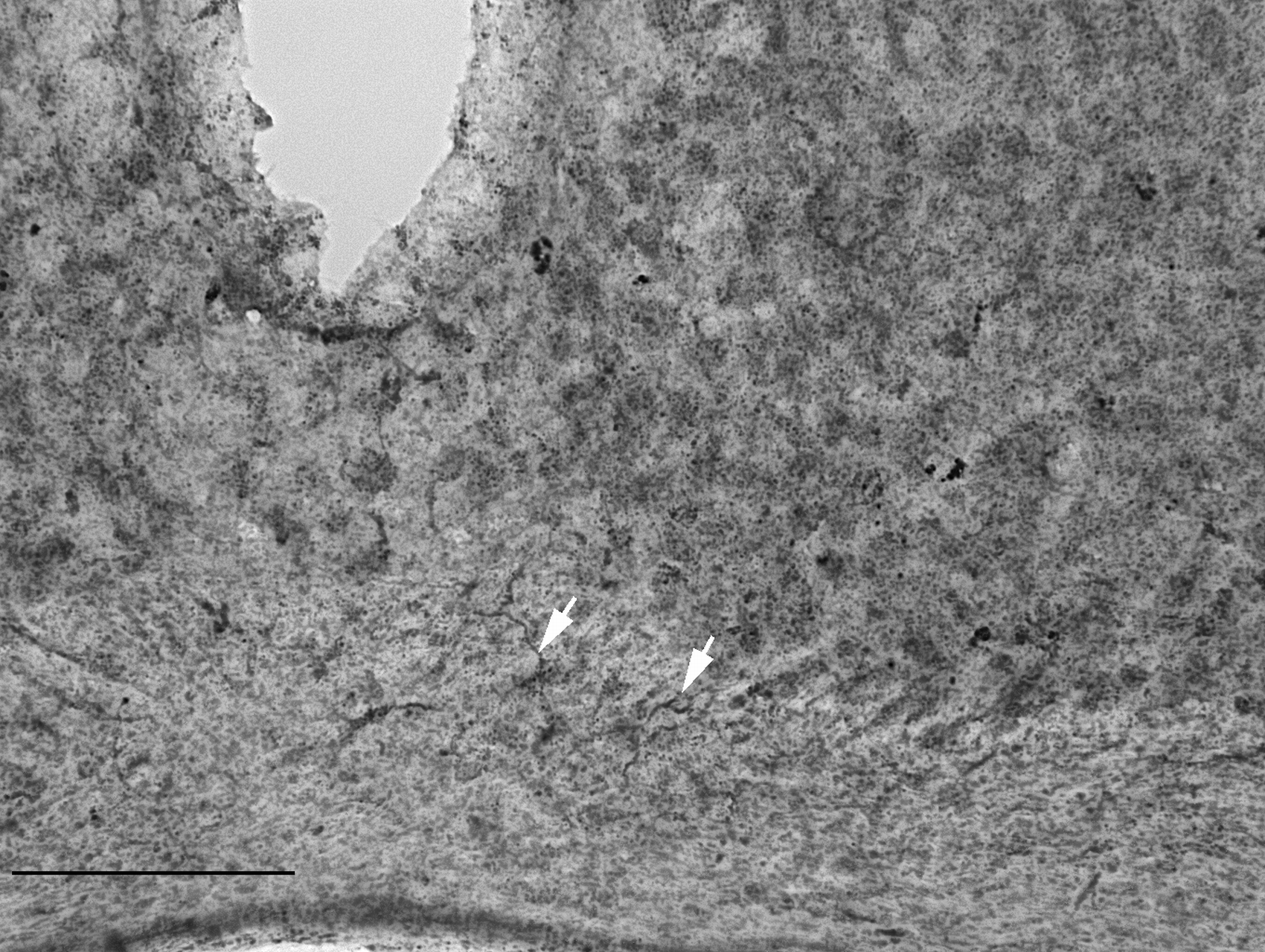

Supplement: Supplementary file 3 — Representative microphotograph of P2X5 immunoreaction in the mouse SCN at high magnification. Mouse was killed at ZT02 (arrows P2X5-immunoreactive astrocyte-processes-like fibres). Bar 25 μm (GIF 2246 kb). [file 441_2017_2634_Fig9_ESM.gif]

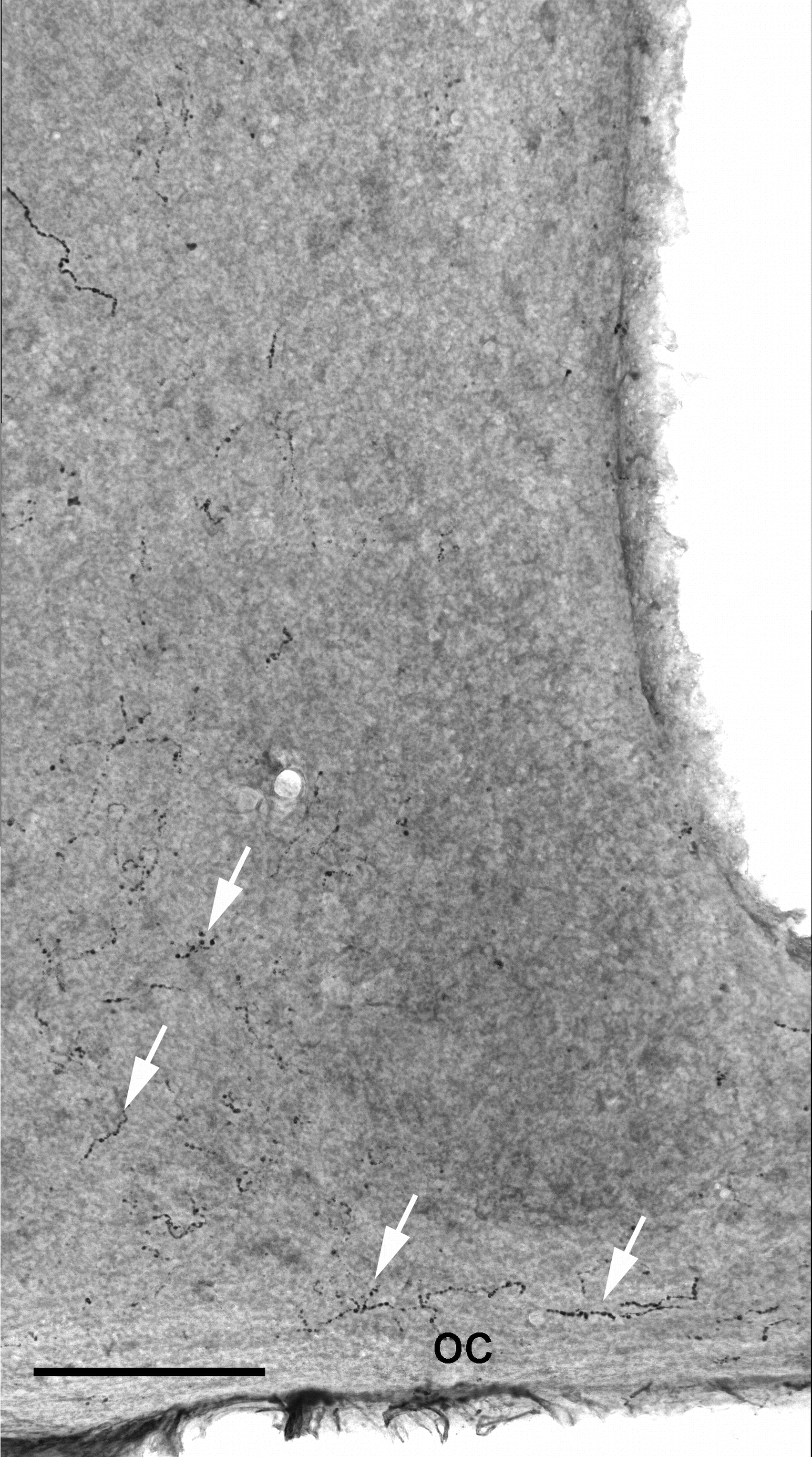

Supplement: Supplementary file 5 — Representative microphotograph of P2Y13 immunoreaction in the mouse SCN at high magnification. Mouse was killed at ZT02 (arrows P2Y13-immunoreactive nerve-ending-like fibres in the core region of the SCN and the optic chiasm [oc]). Bar 100 μm (GIF 1658 kb). [file 441_2017_2634_Fig10_ESM.gif]

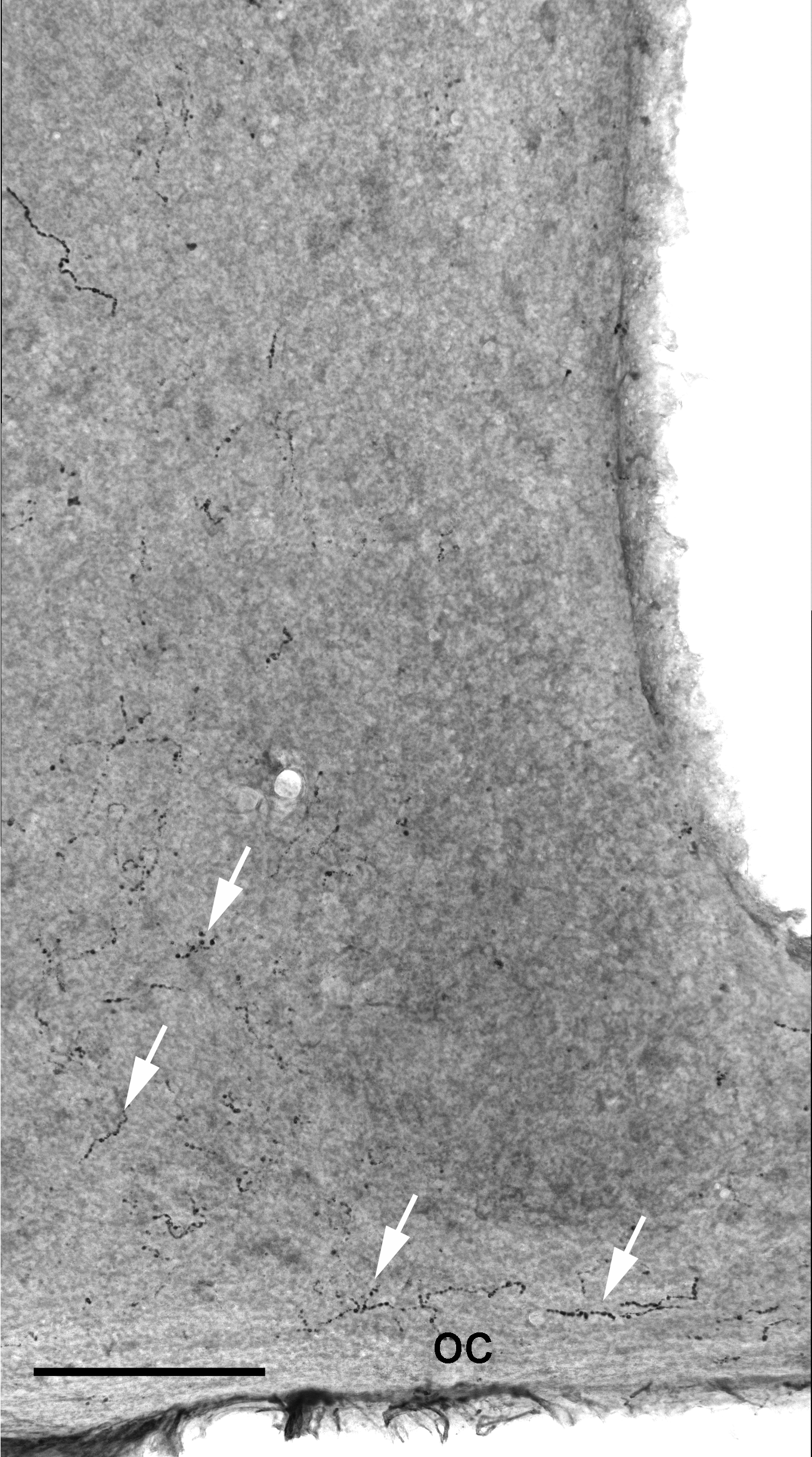

Supplement: Supplementary file 6 — High Resolution Image (TIFF 19082 kb). [file 441_2017_2634_MOESM3_ESM.tif]
